# The Impact of Hepatitis B and/or C on Liver Function and on the Response to Antiretroviral Therapy in HIV-Infected Patients: A Romanian Cohort Study

**DOI:** 10.3390/ph18050688

**Published:** 2025-05-07

**Authors:** Ruxandra-Cristina Marin, Delia Mirela Tit, Gabriela Bungău, Radu Dumitru Moleriu

**Affiliations:** 1 Doctoral School of Biological and Biomedical Sciences, University of Oradea, 410087 Oradea, Romania; marin.ruxandracristina@student.uoradea.ro; 2 Department of Pharmacy, Faculty of Medicine and Pharmacy, University of Oradea, 410028 Oradea, Romania; 3 Department III, Functional Science, Medical Informatics and Biostatistics, University of Medicine and Pharmacy “Victor Babes”, 300041 Timisoara, Romania; radu.moleriu@umft.ro

**Keywords:** HIV, hepatitis B, hepatitis C, coinfections, antiretroviral therapy, immune recovery, liver and pancreatic impairment

## Abstract

**Background**: Hepatitis B (HBV) and C (HCV) virus coinfections remain major contributors to liver-related morbidity and mortality among people living with HIV (PLWH). This study aimed to assess the prevalence of HBV and/or HCV coinfections in a Romanian HIV cohort and to evaluate their impact on immunological, virological, and liver function parameters under antiretroviral therapy (ART). **Methods:** We retrospectively analyzed 462 HIV-infected patients (2018–2021) from the National Institute of Infectious Diseases, Bucharest, stratified into four groups: HIV mono-infection (*n* = 176), HIV/HBV (*n* = 114), HIV/HCV (*n* = 97), and HIV/HBV/HCV (*n* = 75) coinfections. Immunological (CD4 count, CD8 count, and CD4/CD8 ratio), virological (HIV-1 RNA), and hepatic parameters (ALT, AST, GGT, bilirubin, amylase, and lipase) were compared. **Results:** No significant differences were observed between groups regarding the immune recovery (mean CD4 count *p* = 0.89, HIV-RNA suppression *p* = 0.78). However, liver and pancreatic parameters showed statistically significant deterioration in the coinfected groups. ALT (*p* < 0.001), GGT (*p* = 0.009), total bilirubin (*p* = 0.011), amylase (*p* = 0.010), and lipase (*p* < 0.001) were significantly higher in the triple-infection (HIV/HBV/HCV) group compared to HIV mono-infected patients. Coinfection was also associated with a longer duration of illness (*p* = 0.002) and therapy (*p* = 0.021) and with a higher number of ART regimens used (*p* = 0.013). **Conclusions:** While HIV suppression and immune recovery were not significantly impaired by HBV/HCV coinfections, liver and pancreatic injuries were significantly more prevalent and severe in coinfected patients. Regular monitoring of hepatic function and integrated management strategies are recommended to minimize liver-related complications in this population.

## 1. Introduction

The primary causes of liver-related morbidity and mortality among human immunodeficiency virus (HIV)-positive individuals are hepatitis B virus (HBV) and hepatitis C virus (HCV) coinfections. HIV-positive people had a faster natural course of viral hepatitis infection and a faster progression of liver disease to cirrhosis and hepatocellular cancer. Compared to HIV mono-infected groups, HIV/HBV coinfected groups have more hepatotoxicity and a higher median liver enzyme baseline. Compared to HBsAg-negative people, they also have a higher risk of developing liver cirrhosis and fibrosis. Additionally, HCV has a detrimental effect on HIV-positive people, as seen by consistently elevated blood transaminase and an increased incidence of hepatic deterioration [1]. A total of 2.7 and 2.3 million HIV-positive people worldwide have a persistent HBV and HCV coinfection, respectively [2]. Although the prevalence of coinfection varies greatly by area, it is believed that between 8% and 10% of HIV-positive individuals worldwide also have a chronic HBV infection. According to a recent meta-analysis of HBV infection among HIV-positive individuals, the Western Pacific region (11.4%) and sub-Saharan Africa region (10.0%) had the highest frequency of HIV/HBV coinfection, whereas Europe (6.7%) and the Americas (5.3%) had the lowest [3,4].

Those with HIV/HBV coinfection experience a faster development of chronic HBV to cirrhosis, end-stage liver disease, or hepatocellular carcinoma than those with chronic HBV mono-infection. Chronic HBV, on the other hand, has no influence on HIV suppression or CD4 T lymphocyte cell responses when highly active antiretroviral treatment (HAART) is initiated and does not significantly alter the course of HIV infection. Immune reconstitution upon onset or HBV reactivation following cessation of HAART medications that are effective against both HIV and HBV can result in liver-associated problems [5].

The proportion of people living with HIV (PLWH) who are also actively coinfected with HCV was believed to be between 25 and 30 percent prior to the development of direct-acting antivirals (DAA) for the treatment of HCV infection. This percentage was highest among people who injected drugs (PWID) (up to 80%), but it was also high among men who have sex with men (MSM) (3.4%) [6,7].

Coinfection between HCV and HIV is also frequently observed in people who contract HIV through parenteral means [8].

Compared to patients with HIV mono-infection, patients with HIV/HCV coinfection are more likely to experience drug-induced liver damage (DILI) after starting HAART. DILI is most likely to occur in people with advanced liver disease (cirrhosis, end-stage liver disease, etc.) who are also infected with HIV and HCV. Treatment-induced HCV infection eradication reduces the risk of ARV-associated DILI [9,10]. In PLWH coinfected with HCV or/and HBV, levels of aspartate aminotransferase (AST) and alanine aminotransferase (ALT) should be checked 4–8 weeks after starting HAART and then at least every 6–12 months after that, or more frequently if clinically warranted. People with a persistent HCV infection usually have mild to moderate changes in their ALT and/or AST values (less than five times the upper limit of normal [ULN]) [11,12].

Guidelines state that these variations do not justify stopping ART if there are no indications of liver disease or elevated bilirubin, but they do call for monitoring to guarantee a return to baseline. But patients should be closely monitored for signs and symptoms of liver insufficiency, as well as alternative causes of liver injury (e.g., acute HAV or HBV infection, hepatobiliary disease, and alcoholic hepatitis) if they have significant elevations in alanine aminotransferase (ALT) or aspartate transaminase (AST) levels (>5 times IU/L), a concurrent increase in total bilirubin, or concomitant symptoms (weakness, nausea, and vomiting) [5,13]. Thus, the treatment of chronic hepatitis C and B has become a priority in patients with HIV coinfection due to higher survival rates in HIV patients introduced by using of HAART and a quicker onset of liver cirrhosis in HIV patients. Tenofovir alafenamide (TAF) or tenofovir disoproxil fumarate (TDF) with lamivudine (3TC) or emtricitabine (FTC) are indicated as the nucleoside reverse transcriptase inhibitors (NRTI) basis of an antiretroviral (ARV) regimen in HIV/HBV coinfection guidelines [14].

HBV reactivation has been noted following HCV treatment with HAART in patients with HCV/HBV triple-infection. Patients with HIV/HCV coinfection and active HBV infection (HBsAg-positive) should therefore undergo anti-HBV activity-containing ART before starting HCV therapy, along with TDF or TAF with FTC or 3TC [15].

Even in the current HAART age, patients with triple infection of HIV, HCV, and HBV had a 12-fold increased risk of end-stage liver disease compared to patients with HIV mono-infection. Additionally, HIV-positive individuals on HAART who are coinfected with both HBV and HCV have a worse CD4 T-cell recovery and are linked to much higher levels of HIV1-RNA viral load. Furthermore, compared to people with HIV mono-infection, these patients have a higher risk of developing AIDS or dying [16,17].

In Romania, there are now 18,3595 HIV-positive individuals; 60% of them are long-term survivors from the 1990 cohort who were parenterally infected as children [18]. But transmission patterns have changed over time. A significant occurrence in the late 1980s and early 1990s was the high rate of nosocomial infections among children. The F1 HIV-subtype, which was first responsible for HIV transmission through heterosexual contact, was prevalent in this demographic cohort, as well as in adults. That was the beginning of the epidemics in Romania. However, an outbreak among PWID began approximately ten years ago. Additionally, the frequency of HIV diagnoses among men having sex with men (MSM) has steadily increased, according to national data, increasing from roughly 10% of newly reported cases in 2013 to 29% in 2023 [19].

Recent research conducted in Romania provides important light on the incidence of coinfection between HCV and HIV. Between 2018 and 2023, 426,528 individuals who had hepatitis B surface antigen (HBsAg) testing were analyzed in detail. Coinfection rates among the 17,082 HBsAg-positive people (a prevalence rate of 4.0%) were as follows: coinfection rates for HIV and HCV are 0.45% and 1.4%, respectively [20]. Furthermore, 117 coinfected patients were found during published research that focused on people who injected drugs (PWID). Thirteen minor HCV transmission networks and three main HIV clusters were identified using phylogenetic analysis. It is interesting to note that 70 of these patients shared both HIV and HCV clusters, and 80% of these patients were part of both HIV and HCV transmission chains. In 80.3% of cases, HCV infection came before HIV infection, demonstrating the intricate relationship between both illnesses in this group [21].

Given all these factors, the aim of this study was to evaluate the prevalence of HBV/HCV HIV coinfection among a Romanian PLWH cohort, assessing the effect of the coinfections and other possible predictors on the immunologic and virological response to HAART therapy in order of HIV1-RNA viral load (copies/mL) and CD4 lymphocyte T count (cell/mm^3^). Also, this study sought to determine the impact of these coinfections on liver function, taking into consideration and evaluating the values of the hepatic parameters as predictors for the presence or absence of hepatotoxicity.

## 2. Results

### 2.1. Sociodemographic and Clinical Characteristics at Study Inclusion

Considering the sociodemographic and clinical baseline characteristics (Table 1), the statistical analyses showed that for most of the parameters considered, there were no statistical differences between the studied groups. The HIV mono-infection group included 38.10% of the participants, the HIV/HBV coinfection group included 24.64%, the HIV/HCV coinfection group included 21.00%, and the HIV/HBV/HCV triple-infection group contained 16.23% of the entire cohort. The results showed that for age and age at diagnosis, there are no significant difference across the groups (*p* = 0.717, *p* = 0.091, respectively); also, the proportion of males and females is similar (*p* = 0.662). The same statistical results, with no significant differences, were found for the environment and education level and for some parameters describing the clinical characteristics of the immunological and virological baseline HIV infection status: first HIV-1 RNA viral load (copies/mL), CD4 T-cell counts at diagnosis (cells/mm^3^), nadir CD4 T-cell counts, and history of ARVs. The number of years of illness, the number of years of therapy, and the number of ARV regimens show significant differences across the different coinfection groups, with a *p* value of <0.05 (*p* = 0.002, *p* = 0.028, and *p* = 0.017, respectively) for all these parameters.

**Figure 1:**
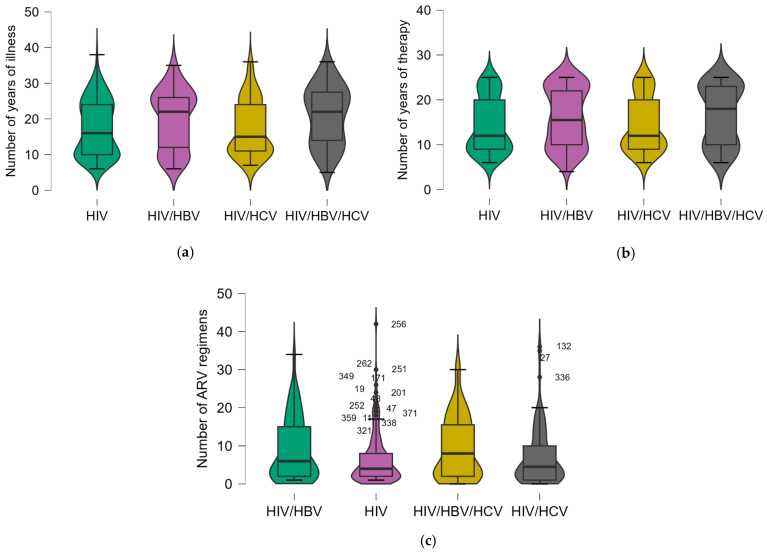
The median value within the studied group for: (**a**) number of years of illness; (**b**) number of years of therapy; (**c**) number of ARV regimens.

The median values for the parameters with statistically significant differences between groups were also represented through a boxplot graphical representation with a violin effect (Figure 1).

For the parameters with statistically significant differences between the groups, a Dunn’s Post Hoc Comparison (Table 2) was run to identify exactly which groups differ significantly from each other. Significant differences were observed in the number of years of illness and therapy between the HIV group and the HIV/HBV/HCV and HIV/HBV groups (*p* = 0.002, *p* = 0.019; *p* = 0.021, *p* = 0.026, respectively), indicating that coinfections impact the disease progression and treatment duration. The number of ARV regimens also showed significant differences, particularly between the HIV group and the HIV/HBV/HCV group (*p* = 0.013), highlighting the complexity of treatment in patients with multiple coinfections. The data show that triple-infections (HIV/HBV/HCV) lead to longer treatment durations and more complex treatment regimens.

### 2.2. Comparative Analysis of Antiretroviral Therapy Regimens and Treatment Response

Table 3 compares the use of various classes of ARV drugs non-nucleoside reverse transcriptase inhibitors (NNRTIs), nucleoside reverse transcriptase inhibitors (NRTIs), protease inhibitors (PIs), integrase inhibitors (IIs), and entry inhibitors (EIs) across the groups. Alongside these drugs, all the participants had a pharmacological enhancer, ritonavir (RTV) or cobicistat (COBI), included in their HIV therapy regimens.

Most participants across all groups used two NRTIs, including 75.57% of HIV, 67.53% of HIV/HBV, 77.30% of HIV/HCV, and 72.00% of HIV/HBV/HCV following this regimen. All the patients from the HIV/HBV and HIV/HBV/HCV groups and 99.41% in HIV and 98.99% in HIV/HCV used at least one PI. A smaller proportion of participants used one II, with the HIV/HBV group showing the highest usage at 25.45%, followed by 21.65% in HIV/HCV, 21.00% in HIV, and 21.30% in HIV/HBV/HCV. As for the use of EI, 1.10% in HIV, 0.88% in HIV/HBV, 1.02% in HIV/HCV, and 0 in HIV/HBV/HCV used one drug from this class.

For all drug classes, no significant differences were found across the four groups, as indicated by the high value of *p*, calculated for each of them.

In the analysis of antiretroviral therapy (ART) regimens (Table 4), no statistically significant differences were observed across study groups regarding the use of NNRTIs (*p* = 0.677), NRTIs (*p* = 0.465), PIs (*p* = 0.637), EIs (*p* = 0.841), or pharmacokinetic boosters (*p* = 0.839). A significant difference was noted for integrase inhibitors (IIs), specifically dolutegravir (DTG) and raltegravir (RAL) (χ^2^ = 12.295, *p* = 0.006).

NRTIs constituted the most frequently utilized class of ARV across all study groups. The combination of abacavir and lamivudine (ABC + 3TC) represented the predominant backbone regimen, particularly in the HIV mono-infected (47.72%) and HIV/HCV coinfected (47.42%) groups. Another commonly prescribed regimen was lamivudine and zidovudine (3TC + ZDV), which was consistently employed across all groups, with notable use in the HIV/HBV and HIV/HCV cohorts (~11%). Furthermore, emtricitabine combined with tenofovir alafenamide or tenofovir disoproxil fumarate (FTC + TAF/TDF) was administered in all groups, although at lower frequencies (~2–3%), and was more frequently observed among patients with HBV or HBV/HCV coinfection, likely reflecting the dual antiviral activity of tenofovir against both HIV and HBV.

NNRTIs were less commonly prescribed in comparison to NRTIs. Etravirine (ETV) was the predominant NNRTI used, with modest representation across all groups, ranging from 10% to 14%. In contrast, efavirenz (EFV) was rarely utilized, appearing in only a single case within the HIV mono-infected group (0.56%), indicating a marked shift away from its use, likely due to concerns regarding neuropsychiatric side effects and lower tolerability.

PIs were a central component of antiretroviral regimens in this cohort. Darunavir (DRV) emerged as the predominant PI, being administered in nearly all patients across all study groups (ranging from 98.97% to 100%), reflecting its high efficacy, resistance barrier, and favorable safety profile. The combination of DRV + atazanavir (ATV) was exceedingly rare, identified in only one case each within the HIV and HIV/HCV groups (0.57% and 1.03%, respectively), underscoring the limited clinical application of dual-PI strategies in current practice.

IIs were variably prescribed across patient groups. Raltegravir (RAL) showed higher prevalence in the HIV (15.34%) and HIV/HBV/HCV (17.33%) groups, reflecting its continued utility in specific clinical scenarios. Dolutegravir (DTG) use was most pronounced in the HIV/HBV group (16.66%), consistent with its potency and resistance profile in HBV coinfection management.

EIs were rarely administered. Maraviroc (MVC) appeared in only four cases across all study groups, indicating its limited role in the treatment landscape, likely due to tropism requirements and niche indications.

Boosters were extensively used to improve the pharmacokinetics of major ARVs. Ritonavir (RTV) was the most common booster, used in 81–85% of patients, whereas cobicistat (COBI) was utilized in a lesser proportion (15–19%), indicating developments in regimen composition and tolerability concerns.

The data in Table 5 reveal the response to therapy among participants with different coinfections, considering various clinical parameters and comparing them across the groups. The *p* value of the mean CD4 T-cell counts, CD8 T-cell counts, and CD4/CD8 ratio across groups indicates no significant difference. Considering the absolute number of the CD4 T-cells, over 50% of the participants, in all studied groups, had counts over 500 cell/mm^3^. CD8 T-cell counts were elevated in the HIV/HCV group compared to other cohorts, while the CD4/CD8 ratio remained relatively consistent across groups, with approximately 25–30% of participants exhibiting a normal ratio greater than 1. The distribution of the participants across groups, by HIV-1 RNA viral load categories, presented no significant difference between the studied groups, with a *p* value of 0.7852. Over 70% of the patients from the HIV mono-infection, HIV/HBV coinfection, and HIV/HBV/HCV triple-infection groups had undetectable viral loads. The lowest percentage (38.00%) in this category was in the HIV/HCV coinfected group. Comparing the stage of the disease, a *p* value of 0.0842 shows no significant differences between groups, though the distribution indicates more advanced disease stages in HIV/HBV/HCV triple-infected groups.

### 2.3. Impact of Antiretroviral Treatment Experience on Immune and Virologic Outcomes

To account for potential differences arising from treatment exposure, we separately analyzed immunological and virological parameters according to ART status. The Mann–Whitney U test was applied for these comparisons due to the non-parametric distribution of the data. Immune and virologic parameters were compared between ART-naïve and ART-experienced patients within each infection group. No statistically significant differences were observed in CD4 T-cell counts, CD8 T-cell counts, CD4/CD8 ratio, or HIV-1 RNA viral load (all *p* values > 0.05, Mann–Whitney U test). This shows that within our cohort, ART treatment history did not significantly influence the immune or virologic parameters at the time of evaluation. Full comparative data are presented in Table 6.

### 2.4. Regression Analysis of Predictive Factors for Immune and Virologic Status

A linear regression model was applied using recent CD4 T-cell count and HIV1-RNA viral load as predictors, with age, age at diagnosis, number of years of illness, number of years of therapy, first RNA (copies/mL), CD4 at diagnosis (cells/mm^3^), and nadir CD4 as the response variable for all four studied groups (HIV, HIV/HBV, and HIV/HCV, respectively, HIV/HBV/HCV).

**Figure 2:**
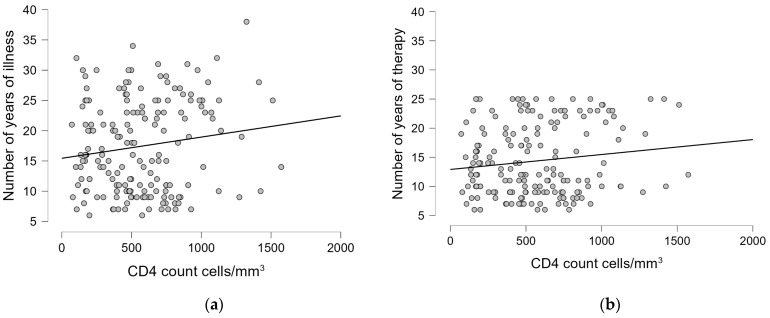
The linear regression graphic showing the correlation between the recent CD4 count cells/mm^3^ and: (**a**) the number of years of illness; (**b**) the number of years of therapy.

For patients with HIV mono-infection, no significant correlations were found for either of the variables (Table 7). For the data that are statistically significant, a graphical representation was made (Figure 2).

For patients with HIV/HBV, no significant correlations were found in most of the cases for CD4 count cells/mm^3^ and HIV1-RNA viral load copies/mL. Between CD4 count, the number of years of illness, and the number of years of therapy, statistically significant but weak negative correlations were observed (r = −0.192, *p* = 0.041; r = −0.197, *p* = 0.035, respectively). While statistically significant, these weak correlations (r^2^ ≈ 0.04) emphasis that only about 4% of the variance in CD4 counts can be explained by these factors, indicating that numerous other clinical variables likely play more substantial roles in determining CD4 counts in these patients. The obtained results are presented in Table 8.

**Figure 3:**
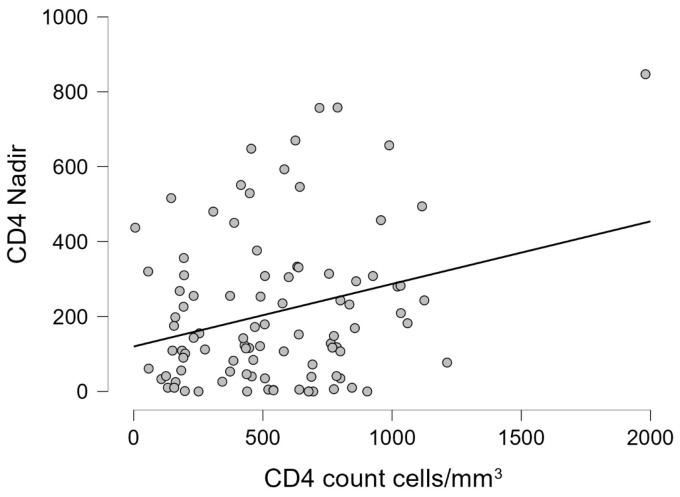
The linear regression graphic showing the correlation between the recent CD4 count cells/mm^3^ and nadir CD4.

For patients with HIV/HCV, no significant correlations were found in either case (CD4 cell count cell/mm^3^ and HIV1-RNA copies/mL), except for a statistically significant but moderate correlation between recent CD4 count and nadir CD4 (r = 0.268, *p* = 0.008). This correlation, while significant, explains only about 7% of the variance (r^2^ = 0.072), indicating that while nadir CD4 have some influence on current CD4 counts, its predictive value is limited, and other factors also substantially influence immune recovery in these patients. (Table 9). For the data that are statistically significant, a graphical representation was made (Figure 3).

**Figure 4:**
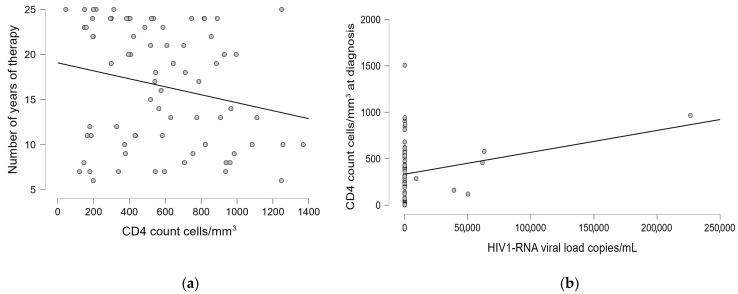
The linear regression graphic showing the correlation between (**a**) the recent CD4 count cells/mm^3^ and the number of years of therapy; (**b**) the recent HIV1-RNA viral load copies/mL and the CD4 count cells/mm^3^ at diagnosis.

For patients with HIV/HBV/HCV, statistically significant but weak correlations were found between recent CD4 count and the number of years of therapy (r = −0.217, *p* = 0.041), and between recent HIV1-RNA viral load and CD4 count at diagnosis (r = 0.229, *p* = 0.049). These weak correlations (r^2^ = 0.047 and r^2^ = 0.052, respectively) indicate that only about 5% of the variance in these outcomes can be explained by these factors. While these associations reached statistical significance, their limited strength shows that they represent modest relationships within a complex clinical picture influenced by numerous other factors. (Table 10). Figure 4 shows the statistically significant graphical representation.

### 2.5. Comparative Analysis of Liver and Pancreatic Function

To evaluate hepatic function, the patients’ liver and pancreatic markers were analyzed, aiming to determine the differences registered between the studied groups (Table 11). Except AST, where there were insignificant differences (*p* = 0.166), in all the other scenarios, the registered differences were statistically significantly different (*p* < 0.001), indicating that the presence of HBV and HCV coinfection induces liver injury and pancreatic dysfunction in HIV patients with those viral infections. Regarding bilirubin (direct, indirect, and total), there was a marked increase in their blood levels, particularly in the HIV/HBV/HCV group. These markers were all significantly higher in coinfected individuals (*p* < 0.001), which indicates more severe liver dysfunction.

**Figure 5:**
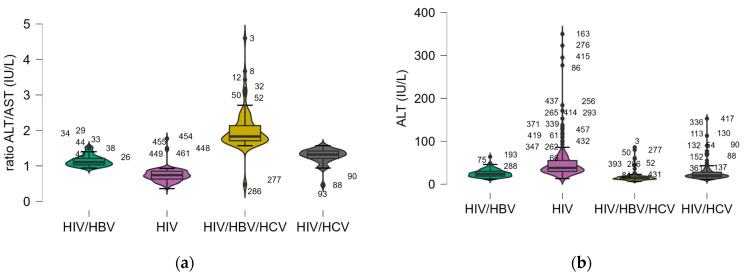

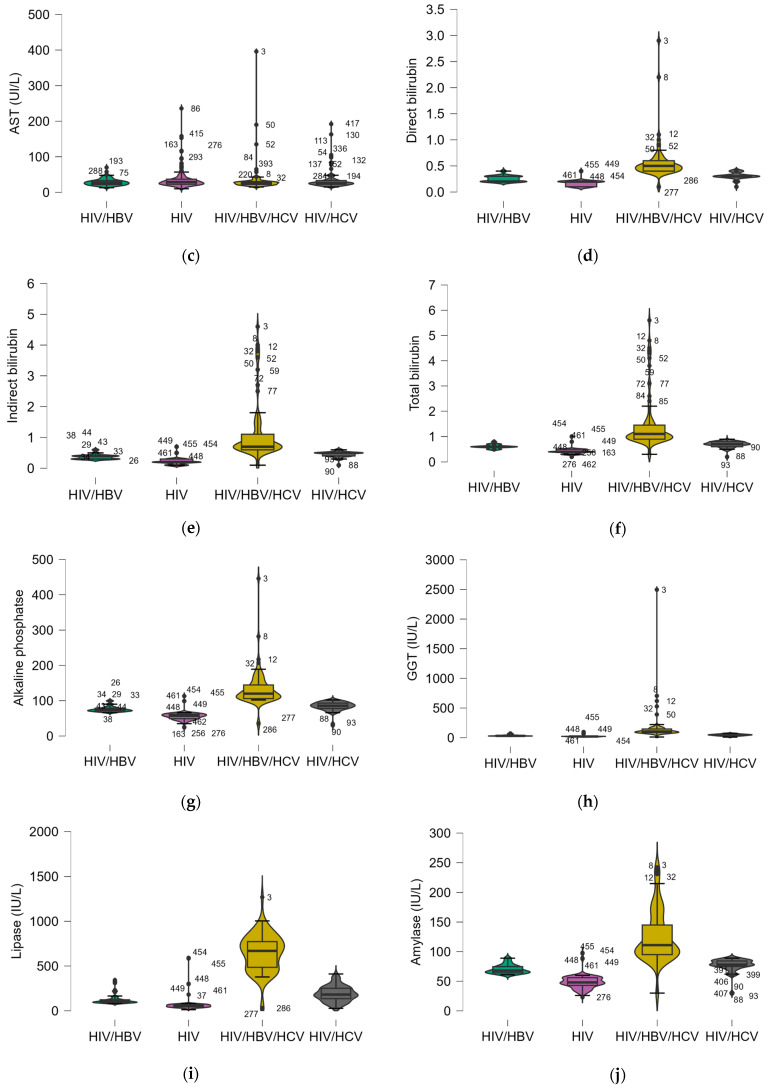
Hepatic parameters median values within the studied groups: (**a**) ALT/AST ratio, (**b**) alanine aminotransferase (ALT), (**c**) aspartate transaminase (AST), (**d**) direct bilirubin, (**e**) indirect bilirubin, (**f**) total bilirubin, (**g**) alkaline phosphatase, (**h**) gamma-glutamyl transferase (GGT), (**i**) lipase, and (**j**) amylase.

To better represent the differences between groups, for the markers with statistically significant differences between groups, a boxplot graphical representation with violin effect was used. The entire analysis is presented in Figure 5.

For the parameters with statistically significant differences between the groups, a Dunn’s Post Hoc Comparison (Table 12) was run to identify exactly which groups differ significantly from each other, focusing on liver function parameters across the different HIV subgroups. Significant differences between coinfected groups (HIV/HBV/HCV, HIV/HCV, and HIV/HBV) were observed across several liver markers, particularly ALT, the AST/ALT ratio, GGT, amylase, bilirubin levels, and ALP (*p* < 0.001). No significant differences in AST and lipase were observed between coinfected groups, which could indicate a more subtle effect of certain coinfections (e.g., HIV/HBV vs. HIV/HCV).

### 2.6. Risk Estimation for Liver Dysfunction Associated with Coinfections

Because coinfections can increase the chance of liver damage, an odds ratio analysis was performed to establish the gravity of the disease and the power of the risk. Being a case–control study, the ODDS ratio parameter and the 95% confidence interval were used to establish the risk. The group of patients having only HIV was considered as a control group, and patients with HBV, HCV coinfection, or triple-infection were the exposed group, resulting in three scenarios that can identify the risks of coinfection in these patients. To determine statistical significance, a Chi-Square Test was applied. The main conclusion is that coinfections can significantly damage (*p* < 0.05) the patients’ health. All the results are presented in Table 13.

## 3. Discussion

This study provides insights into the interplay between HIV infection and coinfections with hepatitis B (HBV) and hepatitis C (HCV), particularly in terms of immune and virological response to HIV therapy, disease progression, and liver function. By evaluating a cohort of Romanian HIV-infected individuals with and without coinfections, this study highlights several important findings when comparing the outcomes of four studied groups: HIV mono-infected, HIV/HBV coinfected, HIV/HCV coinfected, and HIV/HBV/HCV triple-infected.

The sociodemographic characteristics showed no significant differences across groups. These underline a homogenous demographic background and reduce the likelihood that sociodemographic factors confounded our clinical comparisons. However, the age at HIV diagnosis is lower in patients with triple infection (HIV/HBV/HCV) compared to those with HIV mono-infection, though this difference was not statistically significant (*p* = 0.091). A younger age at diagnosis in triple-infected patients reflects earlier or more intensive risk exposures, including behaviors associated with both HIV and viral hepatitis transmission, such as injection drug use or unprotected sexual contact. These results are in line with other studies [22].

When considering the baseline characteristics of the status of the HIV infection, despite the presence of additional coinfections, the viral load at the time of diagnosis, the initial immune status (as indicated by CD4 T-cell counts), and the nadir CD4 count are similar across the groups, demonstrating that coinfection with HBV or HCV does not significantly alter these initial parameters. The immune function at the time of diagnosis might be influenced more by factors other than coinfection with hepatitis viruses. The nadir CD4 count is a critical measure for assessing the severity of HIV infection. Since there are no significant differences, it indicates that coinfections do not dramatically affect the lowest immune function achieved during the course of HIV.

A 2021 study explored how viral hepatitis coinfection affects the response to ART and HIV disease progression. The study highlighted that HBV and HCV coinfections are associated with increased viral load and decreased CD4+ T cell counts in HIV patients. These factors contribute to severe immune suppression, and they necessitate more aggressive management strategies [23].

However, coinfection can lead to a longer duration of illness and more complex treatment regimens. The number of years of illness, the number of years of therapy, and the number of ARV regimens are statistically significantly different across the groups (*p* < 0.05). The outcomes of running the Dunn`s Post Hoc Comparison showed that triple-infection with HBV and HCV leads to longer illness duration, longer therapy durations, and more ARV regimen changes compared to HIV mono-infection or even with HIV/HBV or HIV/HCV coinfection. HIV/HBV coinfected individuals also experience a longer illness duration and more therapy interventions than those with HIV alone, indicating that HBV coinfection has a notable impact on the course of HIV infection. On the other hand, HIV/HCV coinfection did not show a statistically significant increase in illness duration and therapy duration compared to HIV alone, but it still contributes to the complexity of treatment. These findings demonstrate that individuals with more than one coinfection experience earlier onset of chronic conditions or delayed diagnosis, which contributes to extended disease duration. It also shows that people with multiple infections require more time for diagnosis or treatment, potentially leading to a longer overall illness, and coinfections complicate clinical management, delaying therapeutic success [24,25,26].

The analysis of ARV regimens across different studied groups revealed that the treatment regimens are largely similar across all groups. The most used regimens are protease inhibitors (PIs) and nucleoside reverse transcriptase inhibitors (NRTIs), with a significant majority of participants using 2 NRTIs and 1 PI. The use of non-nucleoside reverse transcriptase inhibitors (NNRTIs), integrase inhibitors (IIs), and entry inhibitors (EIs) is much less frequent, and there are no significant differences between the groups in terms of the use of these drug classes. The statistical analysis indicates that there are no significant differences in the therapeutic regimens across the four groups, demonstrating that the presence of coinfections like HBV and HCV does not markedly influence the choice of ARV regimen in this studied population. This uniformity in ARV regimens is indicative of standardized treatment protocols or guidelines followed by healthcare providers across different coinfected and mono-infected patients [25].

The standard of care (SoC) for HIV infected patients in Romania is a triple scheme, which typically consists of a boosted PI, an NRTI, and an NNRTI [26].

Our findings indicate a broad similarity in ART regimen distribution across patient groups, reflecting the standardized application of combination ART (cART) in Romanian clinical practice. The most commonly used regimens were abacavir/lamivudine (ABC + 3TC), darunavir (DRV), and ritonavir (RTV), which is consistent with global patterns preferring NRTIs and protease inhibitors for potency and tolerance. Tenofovir-based combinations (TDF or TAF) were more frequently prescribed to coinfected patients, likely due to their dual efficacy against HIV and HBV, a practice supported by recent studies [24,27].

Integrase inhibitors, particularly dolutegravir (DTG) and raltegravir (RAL), were selectively used, especially among HBV- or HCV-coinfected individuals, aligning with guidelines recommending liver-friendly regimens [25,28]. Recent trials, such as the ALLIANCE study, confirmed the superior efficacy of TAF-based regimens in suppressing HBV replication [28], and broader international data support the increasing adoption of DTG-based therapies for improved safety and lower mortality [29,30].

The reduced use of NNRTIs and the near absence of dual-PI regimens aligns with contemporary practice standards emphasizing better tolerability and resistance profiles [31,32,33]. Our cohort reflects similar prescribing patterns observed across European populations [34], underlining a shift toward simplified, high-barrier ART regimens.

Overall, these findings emphasize the importance of individualized ART selection, particularly in coinfected patients, considering not only regimen potency but also hepatic safety and dual antiviral activity.

ARV therapy is similarly effective across all groups, maintaining relatively stable CD4 counts, CD8 counts, and viral load regardless of whether participants are coinfected with HBV or HCV. Coinfections (HBV and HCV) do not significantly impact the immune parameters or viral load response after therapy. A large proportion of participants in all groups have undetectable HIV-1 RNA, which indicates successful virological suppression. There were no significant differences in disease stage or clinical outcomes across coinfection statuses, indicating that treatment regimens are similarly effective for all participants.

In our study, there were also no significant differences in immune and virologic parameters between ART-naïve and ART-experienced patients (*p* > 0.05), indicating that treatment status did not confound the outcomes in our cohort. Studies before indicated that treatment-naïve individuals showed a stronger and longer-lasting immune response to c-ART, and factors like age, gender, and coinfections play a stronger role in immune recovery than ART experience alone [35,36].

Although coinfected patients had longer HIV infection durations and more complex ART histories, which reflect increased clinical monitoring and comorbidities, similar levels of HIV suppression and CD4 recovery across groups show these variables did not significantly confound our primary findings.

Our results align with studies showing that HBV and HCV coinfections increase treatment complexity without necessarily worsening immune recovery [24].

The correlation coefficients (Pearson’s r) and *p* values for various factors (age, years of illness, therapy, and other clinical variables) against two key health indicators in HIV patients, CD4 count (cells/mm³) and HIV-1 RNA viral load (copies/mL), showed that for patients with mono-HIV infection, there are weak correlations between clinical parameters and immune response, underlining the importance of other factors (like ongoing treatment adherence and regimen) in managing HIV infection. A lack of a significant correlation (*p* = 0.051) between CD4 count and the number of years of illness indicates that there is no clear association between illness duration and CD4 counts in this cohort.

A 2023 meta-analysis examined how low CD4 counts and high HIV viral loads, at the time of diagnosis, affect the efficacy of first-line antiretroviral therapies (ART). The study found that patients with CD4 counts ≤ 200 cells/μL or viral loads ≥ 100,000 copies/mL had increased risks of treatment failure at 48 weeks, with odds ratios of 1.94 and 1.75, respectively [37].

For patients with HIV/HBV coinfection, the negative correlation between the number of years of illness and CD4 count indicates that as the illness duration increases, there is a decrease in immune function (CD4 count cells/mm^3^). Similar results were found in other research. A study observed that HIV/HBV coinfected patients had significantly lower mean CD4 counts compared to HIV mono-infected patients (*p* = 0.014). Additionally, liver enzyme levels (ALT and ALP) were significantly higher in coinfected patients, indicating that a prolonged illness duration contributes to decreased immune function. The authors recommend using non-hepatotoxic antiretroviral drugs for coinfected patients and those with CD4 counts below 200 cells/µL [38].

A longitudinal study found that HIV/HBV coinfected patients had comparable CD4 cell counts and CD4/CD8 ratios to HIV mono-infected patients during pre-ART and follow-up on combined antiretroviral therapy (cART). However, CD4 cell counts were significantly lower in HIV/HCV patients compared to HIV mono-infected patients during follow-up on cART. The study demonstrated that coinfection does not significantly affect the efficacy of cART, but unmeasured factors influence immune response [39].

Overall, the analysis reveals no strong or consistent associations between the studied clinical variables and either CD4 count or HIV-1 RNA levels, underscoring the likelihood that additional, unmeasured factors are influencing immune response and viral load in individuals living with HIV.

For patients with HIV/HCV, factors such as age, age at diagnosis, years of illness, years of therapy, and first RNA levels have no statistically significant correlations with current immune function or viral replication in this cohort. This indicates that other factors, such as viral resistance, treatment adherence, coinfections, or individual biological responses to HIV, play more critical roles in shaping immune response and viral load over time. Nadir CD4 stands out as the most significant predictor of current immune function (CD4 count), with a moderate positive correlation (*p* = 0.008). This shows that the lowest CD4 count ever recorded (nadir) has long-term implications for the current immune status of individuals with HIV.

A descriptive cross-sectional study conducted on 289 seropositive drug-experienced HIV patients, from December 2019 to March 2020, observed that active viral carriers of HBeAg were associated with HBV-HIV coinfected individuals, and HBeAg was associated with severe immune suppression, a decrease in CD4+ T cells, and an increase in viral load. Increased viral load, significant immunological suppression, and a reduction in CD4+ T cells were the most common characteristics in HBV and HCV coinfection in HIV patients [40]. 

As for patients with triple infection (HIV/HBV/HCV), the therapy duration showed a significant negative correlation with CD4 count (*p* < 0.05), indicating that a longer duration of antiretroviral therapy is associated with a lower CD4 count. This highlights the potential challenges in immune recovery or ongoing disease progression despite extended therapy, emphasizing the need for further investigation into factors influencing long-term treatment outcomes. CD4 count at diagnosis was also found to have a significant positive correlation with HIV-1 RNA levels (*p* < 0.05), indicating that patients with a higher initial CD4 count at diagnosis have better viral suppression. This emphasizes the importance of early diagnosis and treatment initiation for better disease management. Other factors did not reach the threshold of statistical significance (*p* < 0.05) when correlated with either CD4 count or HIV-1 RNA levels in this cohort, potentially showing that while these variables play roles in clinical outcomes, they are not directly predictive of immune status or viral load in the studied cohort.

Research in the past few years has reinforced the findings that HIV/HBV/HCV coinfection does not significantly alter the initial immune status or viral load at the time of diagnosis when compared to HIV alone. A study in 2021 found that coinfected patients with HIV/HBV/HCV had comparable initial CD4 counts and viral loads at the time of diagnosis to those with HIV alone, but their disease progression was slower due to delayed diagnosis or more frequent regimen changes [41].

It is important to note that while several statistically significant correlations were identified between clinical parameters in our study, most correlation coefficients were relatively weak (|r| < 0.3). This underlines the fact that while these relationships exist, they explain only a small portion of the variability in immunological and virological outcomes. This finding highlights the complex, multifactorial nature of HIV disease progression and treatment response, particularly in the context of viral coinfections, where numerous host, viral, and treatment factors likely interact to determine clinical outcomes.

HDV coinfection is a known contributor to accelerated liver disease in HBV-infected individuals. A meta-analysis of longitudinal studies found that HBV/HDV coinfected patients have a two-fold increased risk of developing hepatocellular carcinoma (HCC) compared to HBV mono-infected patients. The risk was even higher (six-fold) among HIV/HBV/HDV triple-infected individuals. These findings underscore the accelerated progression of liver disease in the presence of HDV coinfection [42].

However, our study intentionally excluded HDV-positive patients to isolate the impact of HBV and/or HCV coinfections on clinical and laboratory outcomes in people living with HIV. While this approach improved internal consistency, it also limits the generalizability of our findings to patients with triple infection, including HDV. Future studies should include HDV screening and stratification to better capture the full spectrum of liver-related comorbidity in this population.

When analyzing the impact of coinfections on liver and pancreatic function, the data show that the presence of coinfections (HBV and/or HCV) in individuals with HIV significantly increases liver dysfunction, as reflected by hepatic markers (*p* < 0.001). This is particularly evident in ALT, the AST/ALP ratio, GGT, amylase, lipase, and bilirubin levels. Coinfected individuals show elevated markers of liver injury compared to HIV-only patients, indicating more severe liver dysfunction. Patients with HIV/HBV or HIV/HCV coinfections require more intensive treatment regimens to manage liver function and reduce the risk of liver-related complications such as cirrhosis or liver failure. The elevated amylase and lipase levels indicate that pancreatic function is also affected in coinfected individuals (*p* < 0.001). This could be due to the systemic effects of multiple viral infections or side effects from antiviral therapies. Close monitoring of pancreatic enzymes might also be necessary in these cases. These results are consistent with the literature indicating accelerated liver injury, fibrosis, and pancreatic dysfunction in HIV patients with viral hepatitis [43,44,45]. The hepatic burden was more pronounced in patients with triple infection, underscoring the need for intensified monitoring and management.

Hepatotoxicity linked to anti-HIV drugs can exacerbate liver disorders linked to HBV or HCV persistence, whereas HIV-induced immunodeficiency increases the risk of HBV and HCV persistence. Although the exact mechanisms of accelerated hepatocarcinogenesis are still unclear, there is evidence that HIV infection raises the risk of HBV- or HCV-associated hepatocellular carcinoma. Recent progress in eradicating HCV infection and the development of treatment alternatives that effectively suppress both HIV and HBV replication over the long term give hope to patients who are coinfected, but they also underscore the need to choose antiviral medications carefully [46].

A Romanian study comparing the tolerability of two enhanced ARV regimens found that in terms of liver function, patients in the DRV/c group have higher tolerance, as seen in the mean values for ALT, AST, GGT, and ALP, as well as total and direct bilirubin. According to the study, a greater number of individuals who were exposed to RTV as a pharmacokinetic enhancer had elevated parameter values that were directly linked to liver injury. Because it affects drugs’ metabolization process, the enzyme inhibitor plays a relevant role [47].

The findings from Dunn’s Post Hoc Comparisons highlight the significant impact of HIV coinfection with HBV and/or HCV on liver and pancreatic function markers compared to HIV mono-infection. Key liver /biliary and pancreatic enzymes such as ALT, AST/ALT ratio, GGT, amylase, lipase, and bilirubin (both direct and indirect) all show significant differences across the various groups (*p* < 0.001), emphasizing the detrimental effect of coinfections on liver health.

Previous studies on HIV/HBV or/and HCV coinfection have shown mixed results regarding gender differences. A study by Schneider et al. found that women living with HIV/HCV coinfection had worse liver outcomes compared to men, potentially due to differences in immune responses [48].

A retrospective analysis of 394 individuals found that those with chronic hepatitis (7% with HBV and 14% with HCV) were more likely to have elevated liver enzymes than those without coinfection (37% versus 12%, respectively). Transaminases decreased in patients with elevated liver enzymes regardless of whether HAART was maintained or changed. Following the start of HAART, 38% of patients with chronic HBV infection lost HBeAg or acquired anti-HBe, and one seroconverted from HBsAg-positive to anti-HBs-positive. The conclusion was that in contrast to individuals without coinfection, HIV-1-infected patients who were also coinfected with HBV or HCV had a significantly increased risk of developing elevated hepatic markers, and there is no need to change the ARV regimen [49].

There are studies that state that the type of ARV used can determine when liver damage will occur. Nucleoside analogues cause hepatic steatosis, most likely due to mitochondrial toxicity, within six months of treatment, according to a study by Carr et al. [50]. After being administered for three months, NNRTIs seem to result in hypersensitivity reactions. For patients who already have liver disease, liver function tests should be performed every three months; for those who do not, tests should be performed monthly.

For patients diagnosed with liver disease, regular monitoring is crucial to assess disease progression and treatment efficacy. The World Health Organization (WHO) recommends at least annual monitoring of liver function in individuals with chronic hepatitis B, including assessments of alanine aminotransferase (ALT), hepatitis B e antigen (HBeAg), and hepatitis B virus (HBV) DNA levels. More frequent monitoring is necessary for those with fluctuating ALT or HBV DNA levels, or those with coinfection with HIV, who should be monitored every 6 to 12 months. In the absence of clinical symptoms, treatment can continue under monitoring if the liver enzyme values are within the moderately high range, less than 3.5 times the normal upper limit, of ALT-30 IU/L and AST-25 IU/L [51].

For patients without known liver disease, the frequency of liver function test (LFT) monitoring is less well-defined. However, clinical guidelines show that in the absence of symptoms or known liver disease, there is no need for frequent monitoring. The British Society of Gastroenterology notes that liver disease often develops silently, and routine liver blood tests should be considered to determine the presence or severity of liver disease, especially in individuals with risk factors or nonspecific symptoms [52].

Increased liver enzyme levels need additional testing, such as imaging and measuring drug plasma levels. Treatment can continue under observation if the liver enzyme values are within the moderately high range, less than 3.5 times the usual upper limit, and if there are no clinical complaints. But additional investigations, such as imaging and drug plasma concentration measurement, are necessary for higher elevations in liver enzymes. Studies underline that particular treatment for viral hepatitis infections should also be considered for individuals with HBV and/or HCV, since these coinfections decrease the efficiency of ART and increase liver toxicity [53].

According to previous research, pancreatic disorders—typically pancreatitis—or extra-pancreatic diseases, such as renal, acidosis, parotid, or gastrointestinal disorders, are the causes of hyperamylasemia and hyperlipasemia. Pancreatitis and the development of pancreatic enzymes are not related. HIV+ individuals frequently experience mild to moderate elevations, which have been linked to various medications, particularly intravenous Cotrimoxazole or antiretroviral medications, as well as positive serology results for chronic B or C hepatitis. Elevated blood levels of lipase and amylase have also been found in numerous investigations [54].

Analyzing the power of the risk of coinfections, the outcomes showed that the odds ratios for liver function markers like ALT, AST, GGT, amylase, and lipase are consistently high, indicating a strong likelihood of liver dysfunction in the coinfected groups. Additionally, parameters such as the AST/ALT ratio and bilirubin levels further emphasize the presence of significant liver damage in these patients.

Overall, these findings underscore the need for regular monitoring and management of liver function in HIV patients, especially in patients coinfected with HBV and/or HCV. Clinicians should be careful in managing these individuals to prevent further liver deterioration and to guide appropriate treatment strategies. Early intervention and close follow-ups are crucial for improving patient outcomes and minimizing the risk of severe liver-related complications.

The limitations of this study refer to its retrospective nature. We could not include some relevant clinical data referring to markers of hepatitis virus infection: markers for HBV coinfection (serum HBsAg, anti-HBc IgM and total, and HBV viral DNA) and markers for HCV coinfection (HCV viral RNA, anti-HCV antibodies). The presence or absence of abnormal liver function was not confirmed by more precise diagnostic tests, such as liver biopsy and ultrasonography, in patients with high amounts of liver enzymes. This could have led to an underestimation of the degree of liver fibrosis, steatosis, or cirrhosis, as supported by previous studies showing that biochemical markers alone may not fully capture liver damage severity [55,56,57,58]. Moreover, the absence of viral quantification limited our ability to explore associations between viral loads and liver dysfunction, although prior research has shown that high HBV or HCV loads correlate with worse outcomes [55,56]. We also lacked information on whether HBV and HCV infections occurred simultaneously with or after HIV infection, a factor that may influence disease progression and immune recovery, as highlighted in earlier studies [59,60].

Another important limitation was the absence of data on concomitant medications. Several commonly prescribed drugs for HIV comorbidities—such as antimycobacterials, azole antifungals, and antibiotics—are known to cause drug-induced liver injury (DILI) [61,62,63], potentially confounding the observed liver abnormalities. Although we excluded patients with documented non-viral liver disease, we cannot entirely rule out unrecognized cases of fatty liver disease or alcohol-related liver damage, which are prevalent in people living with HIV [64,65]. Despite these limitations, our findings offer valuable real-world insights into the management of HIV and viral hepatitis coinfections, particularly in resource-limited settings. Future prospective studies should incorporate viral load monitoring, infection chronology, medication history, and more definitive assessments of liver disease to better understand the interplay between these infections and their clinical outcomes.

## 4. Materials and Methods

### 4.1. Study Design

This retrospective observational study enrolled consecutively HIV-infected patients with complete clinical, virological, immunological, and hepatic/pancreatic biomarker data from the institutional records during the study period (October 2018–December 2021). From the sample size formula and the post hoc power analysis, it was determined that the sample size of 462 patients provided sufficient power (>80%) to detect statistically significant differences in hepatic parameters when assuming a moderate effect size (Cohen’s d = 0.5) for differences in liver enzyme levels (e.g., ALT) between HIV mono-infected (*n* = 176) and HIV/HBV/HCV triple-coinfected groups (*n* = 75). The achieved power exceeded 95% at a two-sided alpha level of 0.05. This demonstrates that the study has the power to detect clinically meaningful differences in hepatic function.

The analyzed data were collected from routine clinical assessments and were retrospectively extracted from archived medical records.

Patients were selected according to eligibility criteria, which included HIV-infected patients, naïve or experienced, on a stable ART regimen for a minimum of six months prior to enrollment, over 18 years old, regardless of HIV-RNA viral load and the CD4 and CD8 T lymphocyte count. In order to be included, patients had to have all the necessary information for analysis in their medical records, including HIV1-RNA viral load (copies/mL); levels of CD4 and CD8 T cells/mm^3^; HBV and/or HCV coinfection status; and laboratory results of the main hepatic and pancreatic biomarkers. According to their antiretroviral therapy (ART) status, experienced patients were considered those with more than one regimen in their therapeutic history, and naive patients were considered those who had recently initiated ART, being, at the time of the study’s start, in their first ART regimen, but for at least six months.

Patients under 18 years old, pregnant or breastfeeding women, patients with other medical conditions that could affect the results, as well as patients with incomplete data on immunological and virological parameters or viral coinfections, were excluded from the study. Also, participants who had recently initiated ART (within less than six months) were excluded to minimize variability related to early treatment effects.

Patients with confirmed or suspected HDV coinfection, based on available serological or molecular testing, were excluded from this analysis to ensure homogeneity within the HBV coinfection group. Participants with known or suspected non-viral liver diseases—including alcoholic liver disease, NAFLD, autoimmune hepatitis, or biopsy-confirmed cirrhosis of non-viral origin—were also excluded based on clinical history or previous diagnoses recorded in the medical files. Individuals with a documented history of alcohol use disorder were also excluded. Only patients with HBV and/or HCV-related liver involvement and laboratory data were retained in the analysis.

The cohort was divided into four study groups, according to the presence or absence of HCV or/and HBV coinfection: HIV mono-infected group—176 (38.10%), HIV/HCV coinfection group—97 (21.00%), HIV/HBV coinfection group—114 (24.67%), and triple-coinfection (HIV/HCV/HBV) group—75 (16.23%).

This study was approved by the Manager of NIID MB (approval No. 9/5861 of 4 May 2021) and by the same institution’s Bioethics Committee (approval No. C05865 of 4 May 2021). The Ethical Commission of the Faculty of Medicine and Pharmacy from University of Oradea also approved this research. This study was carried out in compliance with the Good Clinical Practice guidelines and the ethical standards outlined in the Helsinki Declaration [66,67].

The data were sourced from anonymous, non-personally recognizable medical records. Given that the analysis was based exclusively on retrospective and anonymized data, without interventions or direct contact with patients, the Bioethics Committee granted permission to use the data without individual informed consent (waiver of informed consent).

### 4.2. Clinical and Biochemical Determinations

Retrospective examination of biochemical and clinical, immunological, and virological data from archived medical records was used to evaluate the patients. Absolute CD4 and CD8 T-lymphocyte counts, CD4/CD8 ratio, and plasma HIV-1 RNA viral load were among the relevant parameters. Using tools from the NIID MB Immunology laboratory, the number of CD4 and CD8 T-cells was determined using flow cytometry. Cells/mm^3^ were used to express the values. By dividing the absolute value of CD4 cells by the value of CD8 lymphocytes, the CD4/CD8 ratio was mathematically calculated. A ratio greater than 1 was regarded as normal, and a ratio less than 1 was regarded as inverted [68].

Using real-time polymerase chain reaction (PCR) in accordance with a standardized internal technique, the HIV-1 RNA virus load was measured. During routine clinical visits, plasma samples from venous blood taken in EDTA vacutainers were used for the analyses [69].

The Center for Disease Control and Prevention (CDC) class guide was used to determine the illness stage based on the level count of CD4/mm^3^ blood cells [70]. The immunological and virological profiles of the four groups, depending on coinfection status, were compared using cross-sectional laboratory data analysis.

The biochemical indicators included in the study were evaluated in ordinary clinical practice. Laboratory parameters were selected based on the values obtained closest to the start of the study period to ensure a consistent and standardized baseline across all participants. The blood samples were collected in the morning after a 12–14 h meal break. Serum levels of liver enzymes (alanine aminotransferase—ALT/GPT, aspartate aminotransferase—AST/GOT, gamma-glutamyl transferase—GGT, alkaline phosphatase—ALP, total bilirubin, direct bilirubin) and pancreatic enzymes (amylase and lipase) were collected and analyzed to determine hepatic and pancreatic function.

In our study, HBV and HCV coinfection status was determined based on documented diagnoses in the existing medical records at NIID MB. These diagnoses followed the European guidelines for viral hepatitis management, which align with European Association for the Study of the Liver (EASL) recommendations [70].

According to the same guidelines, HBV infection diagnosis requires detection of hepatitis B surface antigen (HBsAg) using enzyme-linked immunosorbent assay (ELISA). Patients were classified as having HBV coinfection if they had a documented positive HBsAg test in their medical records. At NIID MB, the standard protocol involves confirmatory testing with additional markers (HBeAg, anti-HBe, antiHBc IgM, and total HBV DNA) for clinical management, but for our study classification, a documented positive HBsAg test was considered sufficient for inclusion in the HBV coinfected groups.

HCV infection diagnosis was established by detection of anti-HCV antibodies by ELISA, with confirmation via HCV RNA testing for active infection, according to the international guidelines [70].

Patients were classified as having HCV coinfection if they had a documented positive anti-HCV antibody test in their medical records. While HCV RNA quantification is performed for clinical management at NIID MB, for study classification purposes, a documented positive anti-HCV antibody result was used as the inclusion criterion for the HCV coinfected groups. The laboratory uses third-generation ELISA tests with sensitivity and specificity exceeding 99% for both HBsAg and anti-HCV detection. As noted in our limitations section, we did not have access to complete data on all serological and molecular markers for all patients, which represents a limitation of our retrospective study design. However, the classifications used reflect standard diagnostic practice in Romania during the study period.

### 4.3. Statistical Analysis

The data were gathered in a Microsoft Excel file and statistically processed using the JASP v19.3 program. A descriptive analysis was applied, starting with the data distribution, where a Shapiro–Wilk test was applied for all numerical variables, concluding that a *p* value over 0.05 determines a normal distribution. In most cases, the data in our study are not normally distributed (*p* < 0.05). The entire analysis was run between these four groups. Descriptive analysis was completed to describe the data and to test its distribution. Due to the non-normal distribution of data (as verified by the Shapiro–Wilk test), we used non-parametric methods. To test the existing possible differences within the four groups, a Kruskal–Wallis test was applied for continuous parameters, followed by Dunn’s post hoc tests for pairwise comparisons. Dunn’s Post Hoc Comparisons is based on individual Mann–Whitney tests and biserial correlations, and it was used to see what happens between the studied groups. Bonferroni correction was applied to adjust for multiple testing in these post hoc analyses.

A Chi-Squared (x^2^) Test was run to determine categorical parameters. For correlation analyses, both Pearson’s correlation coefficient (r) and the coefficient of determination (r^2^) were calculated to assess the strength and practical significance of relationships between variables. While the *p* value determines statistical significance (set at α = 0.05), the r^2^ value quantifies the proportion of variance in one variable that can be explained by the other variable. Correlations were categorized as very weak (|r| < 0.2, r^2^ < 0.04), weak (|r| = 0.2–0.4, r^2^ = 0.04–0.16), moderate (|r| = 0.4–0.6, r^2^ = 0.16–0.36), strong (|r| = 0.6–0.8, r^2^ = 0.36–0.64), and very strong (|r| > 0.8, r^2^ > 0.64). This classification helps contextualize the practical significance of statistically significant correlations in clinical interpretation. To adjust for potential confounding, correlation analyses included the following covariates: age, sex, age at HIV diagnosis, duration of infection, duration of ART, number of ART regimens used, baseline HIV1-RNA load, CD4 count at diagnosis, and nadir CD4.

To determine possible associations between data, linear regression and a multiple linear regression analysis were performed. In the end, odds ratio analysis models were employed to assess the association between viral coinfection (HIV/HBV, HIV/HCV, or HIV/HBV/HCV) and abnormal hepatic and pancreatic biomarkers. Odds ratios (ORs) with 95% confidence intervals (CIs) were calculated to quantify the strength of association. A *p* value < 0.05 was considered statistically significant. A *p* value < 0.05 was considered statistically significant. The level of confidence was set at α=0.05.

## 5. Conclusions

This study demonstrates that HIV treatment remains highly effective in controlling HIV replication, restoring immune function, and preventing disease progression, even in the presence of HBV and HCV coinfections. Coinfection does not appear to significantly alter the therapeutic response, and ART should remain the cornerstone of treatment in these patients. However, ongoing research is essential to fully understand the long-term implications of coinfection and the optimal management strategies for this complex patient population. Despite limitations, the findings offer valuable insight into the complexities of managing coinfected populations in routine clinical settings. The observed variations in liver function parameters and immune markers highlight the need for integrated monitoring strategies that consider not only viral interactions but also the broader context of polypharmacy and comorbidity management. Future research should aim to address these gaps through longitudinal designs and detailed therapeutic profiling to better delineate the multifactorial influences on immune recovery and hepatic outcomes in coinfected individuals. Our findings underscore the necessity of individualized, multidisciplinary approaches to optimize long-term care in this vulnerable population.

## Figures and Tables

**Table 1:** Baseline sociodemographic and clinical characteristics of the participants.

Parameters		HIV *n* = 176	HIV/HBV *n* = 114	HIV/HCV *n* = 97	HIV/HBV/HCV *n* = 75	Statistics	*p* Value
Age (years, mean ± SD)		46.006 ± 11.750	45.053 ± 10.865	44.371 ± 9.817	45.067 ± 12.823	1.352 ^1^	0.717 ^1^
Age at diagnosis (years, mean ± SD)		28.540 ± 13.271	25.298 ± 13.120	27.093 ± 11.476	24.160 ± 14.802	6.476 ^1^	0.091 ^1^
Sex *n* (%)	Male	105 (59.66)	76 (66.67)	60 (61.86)	45 (60.00)	1.588 ^2^	0.662 ^2^
	Female	71 (40.34)	38 (33.33)	37 (38.14)	30 (40)		
Environment *n* (%)	Rural	48 (27.27)	28 (24.56)	18 (18.56)	23 (30.67)	3.869 ^2^	0.276 ^2^
	Urban	128 (72.73)	86 (75.44)	79 (81.44)	52 (69.33)		
Education level *n* (%)	Illiterate	3 (1.70)	2 (1.75)	2 (2.06)	2 (2.67)	25.556 ^2^	0.110 ^2^
	High school	99 (56.25)	74 (64.91)	61 (62.89)	49 (65.33)		
	Professional	7 (3.97)	14 (12.28)	11 (11.34)	8 (10.67)		
	University	35 (19.89)	12 (10.53)	7 (7.22)	7 (9.33)		
	Unknown	32 (18.18)	12 (10.52)	16 (16.50)	9 (12)		
No. of years illness (mean ± SD)		17.466 ± 7.612	19.754 ± 8.204	17.278 ± 7.670	20.907 ± 8.128	14.559 ^1^	0.002 ^1^
No. of years of therapy (mean ± SD)		14.381 ± 6.079	16.088 ± 6.382	14.351 ± 6.093	16.547 ± 6.595	9.126 ^1^	0.028 ^1^
First HIV-1 RNA viral load (copies/mL) (mean ± SD)		513,307.858 ± 1.545 × 10^+6^	317,901.579 ± 866,724.689	397,283.186 ± 1.270 × 10^+6^	1.150 × 10^+6^ ± 132,771.892	3.532 ^1^	0.317 ^1^
CD4 T-cell counts at diagnosis (cells/mm^3^) (mean ± SD)		333.443 ± 254.257	372.737 ± 256.764	369.928 ± 361.502	294.360	2.138 ^1^	0.544 ^1^
Nadir CD4 T-cell counts (cells/mm^3^) (mean ± SD)		213.591 ± 186.106	214.509 ± 182.966	208.464 ± 203.001	175.360 ± 159.206	2.470 ^1^	0.481 ^1^
History of ARVs *n* (%)	Naive	9 (5.11)	6 (5.27)	11 (11.34)	4 (5.33)	4.755 ^2^	0.191 ^2^
	Experienced	167 (94.89)	108 (94.73)	86 (88.66)	71 (94.67)		
No. of ARV regimens (mean ± SD)		6.506 ± 6.524	9.018 ± 8.042	7.041 ± 7.331	9.480 ± 8.026	10.143 ^1^	0.017 ^1^

*n*—number of participants; No.—number; mL—milliliter; mm^3^—cube millimeter; SD—standard deviation; ARV—antiretroviral drugs; ^1^—Kruskal–Wallis Test; ^2^—Chi-Squared Test.

**Table 2:** Dunn’s Post Hoc Comparisons between studied groups.

Group	Comparison	No. of Years Illness/ *p* Value	No. of Years Therapy/ *p* Value	No. of ARV Regimens
HIV	HIV/HBV/HCV	0.002	0.021	0.013
	HIV/HCV	0.927	0.921	0.869
	HIV/HBV	0.019	0.026	0.019
HIV/HBV/HCV	HIV/HCV	0.004	0.046	0.036
	HIV/HBV	0.299	0.728	0.682
HIV/HCV	HIV/HBV	0.034	0.065	0.059

**Table 3:** Descriptive statistics of the therapeutic ARV regimens, according to the ARV classes.

Parameters in *n* (%)		HIV *n* = 176	HIV/HBV *n* = 114	HIV/HCV *n* = 97	HIV/HBV/HCV *n* = 75	Statistics	*p* Value
NNRTI	None	148 (84.09)	96 (21.00)	86 (88.65)	63 (88.66)	1.251 ^2^	0.741 ^2^
	1	28 (15.91)	18 (15.79)	11 (11.34)	12 (16.06)		
NRTI	None	22 (12.52)	14 (12.28)	8 (8.25)	8 (10.65)	15.504 ^2^	0.215 ^2^
	1	19 (10.83)	21 (18.42)	9 (9.28)	11 (14.67)		
	2	133 (75.57)	77 (67.53)	75 (77.30)	54 (72.00)		
	3	2 (1.14)	1 (0.88)	2 (2.06)	2 (2.62)		
	4	0 (0.00)	1 (0.88)	3 (3.12)	0 (0.00)		
PI	None	0 (0.00)	0 (0.00)	0 (0.00)	0 (0.00)	1.701 ^2^	0.637 ^2^
	1	175 (99.41)	144 (100.00)	96 (98.99)	75 (100.00)		
	2	1 (0.57)	0 (0.00)	1 (1.03)	0 (0.00)		
II	None	138 (78.43)	85 (74.56)	76 (78.34)	59 (78.67)	2.475 ^2^	0.871 ^2^
	1	37 (21.00)	29 (25.45)	21 (21.65)	16 (21.30)		
EI	None	174 (98.90)	113 (99.13)	96 (98.97)	75 (100.00)	0.836 ^2^	0.841 ^2^
	1	2 (1.10)	1 (0.88)	1 (1.02)	0 (0.00)		

*n*—number; NNRTI—non-nucleoside reverse transcriptase inhibitor; NRTI—nucleoside reverse transcriptase inhibitor; PI—protease inhibitor; II—integrase inhibitor; EI—entry inhibitor, ^2^—Chi-Squared Test.

**Table 4:** Descriptive statistics of the therapeutic ARV regimens, according to the ARV types.

Parameters in *n* (%)		HIV *n* = 176	HIV/HBV *n* = 114	HIV/HCV *n* = 97	HIV/HBV/HCV *n* = 75	Statistics	*p* Value
NNRTI types	EFZ	1 (0.56)	0 (0.00)	0 (0.00)	0 (0.00)	1.523 ^2^	0.677 ^2^
	ETV	25 (14.20)	17	11 (11.34)	11 (14.66)		
NRTI types	3TC	2 (1.13)	0 (0.00)	0 (0.00)	3 (4.00)	45.159 ^2^	0.465 ^2^
	3TC + TDF	0 (0.00)	0 (0.00)	1 (1.03)	0 (0.00)		
	3TC + ZDV	18 (10.22)	13 (11.40)	11 (11.34)	9 (12.00)		
	3TC + ZDV + FTC + TDF	0 (0.00)	1 (0.88)	0 (0.00)	0 (0.00)		
	ABC	1 (0.57)	0 (0.00)	0 (0.00)	0 (0.00)		
	ABC + 3TC	84 (47.72)	43 (37.71)	46 (47.42)	33 (44.00)		
	ABC + 3TC + TDF	1 (0.57)	0 (0.00)	0 (0.00)	2 (2.66)		
	ABC + 3TC + ZDV	1 (0.57)	0 (0.00)	2 (2.06)	0 (0.00)		
	ABC + TDF	0 (0.00)	1 (0.88)	1 (1.03)	0 (0.00)		
	FTC	3 (1.70)	0 (0.00)	2 (2.06)	0 (0.00)		
	FTC + TAF	5 (2.84)	4 (3.51)	2 (2.06)	2 (2.66)		
	FTC + TDF	5 (2.84)	4 (3.51)	2 (2.06)	2 (2.66)		
	TDF	13 (7.39)	18 (15.79)	7 (7.22)	11 (14.66)		
	TDF + 3TC	1 (0.57)	0 (0.00)	0 (0.00)	0 (0.00)		
	FTC + TAF + ABC + 3TC	25 (14.20)	17 (14.91)	16 (16.49)	10 (13.33)		
PI types	DRV	175 (99.43)	114 (100.00)	96 (98.97)	75 (100.00)	1.701 ^2^	0.637 ^2^
	DRV + ATV	1 (0.57)	0 (0.00)	1 (1.03)	0 (0.00)		
II types	DTG	12 (6.82)	19 (16.66)	8 (8.24)	3 (4.00)	12.295 ^2^	0.006 ^2^
	RAL	27 (15.34)	10 (8.77)	13 (13.40)	13 (17.33)		
EI types	MVC	2 (1.13)	1 (0.88)	1 (1.03)	0 (0.00)	0.836 ^2^	0.841 ^2^
Booster	RTV	143 (81.25)	95 (83.33)	82 (84.53)	64 (85.33)	0.843 ^2^	0.839 ^2^
	COBI	33 (18.75)	19 (16.66)	15 (15.46)	11 (14.66)		

ABC—abacavir; COBI—cobicistat; DTG—dolutegravir; DRV—darunavir; EFZ—efavirenz; ETV—etravirine; FTC—emtricitabine; *n*—number; RAL—raltegravir; RTV—ritonavir; TAF—tenofovir alafenamide; TDF—tenofovir disoproxil fumarate; ZDV—zidovudine; 3TC—lamivudine, ^2^—Chi-Squared Test.

**Table 5:** Clinical characteristics of the participants representing the response to the present therapy.

Parameters			HIV *n* = 176	HIV/HBV *n* = 114	HIV/HCV *n* = 97	HIV/HBV/HCV *n* = 75	Statistics	*p* Value
CD4 T-cell counts (cells/mm^3^) (mean ± SD)			563.114 ± 320.227	571.386 ± 302.299	545.928 ± 325.586	570.387 ± 324.183	0.622 ^1^	0.891 ^1^
CD8 T-cell counts (cells/mm^3^) (mean ± SD)			862.241 ± 423.662	909.728 ± 391.282	925.361 ± 610.541	764.653 ± 357.669	6.865 ^1^	0.076 ^1^
CD4/CD8 Ratio (mean ± SD)			0.848 ± 0.727	0.757 ± 0.574	0.962 ± 1.859	0.888 ± 0.699	2.344 ^1^	0.504 ^1^
CD4 cell counts categories *n* (%)	0–199		33 (18.75)	21 (18.42)	21 (21.65)	15 (20.00)	5.108 ^2^	0.825 ^2^
	200–349		13 (7.40)	8 (7.02)	7 (7.22)	7 (9.33)		
	350–499		40 (22.72)	17 (14.91)	20 (20.62)	11 (14.67)		
	>500		90 (51.14)	68 (59.65)	49 (50.51)	42.(56.00)		
CD4/CD8 ratio categories *n* (%)		≤0.50	51 (29.82)	42 (40.54)	35 (36.08)	30 (40.54)	5.584 ^2^	0.471 ^2^
		0.51–0.99	76 (44.44)	38 (33.62)	38 (39.17)	24 (32.43)		
		≥1	44 (27.73)	33 (29.20)	24 (24.74)	20 (27.02)		
HIV-1 RNA viral load categories *n* (%)	0		133 (75.56)	87 (76.32)	77 (79.38)	63 (84.00)	5.544 ^2^	0.785 ^2^
	<50		1 (0.57)	0 (0.00)	0 (0.00)	0 (0.00)		
	50–999		24 (13.63)	17 (14.91)	13 (13.40)	5 (6.67)		
	˃1000		18 (10.22)	10 (8.77)	7 (7.21)	7 (9.33)		
Stage of disease *n* (%)	A1		6 (3.40)	3 (2.63)	2 (2.06)	0 (0.00)	34.040 ^2^	0.084 ^2^
	A2		22 (12.50)	4 (3.50)	5(5.15)	2 (2.67)		
	A3		6 (3.40)	1 (0.88)	3 (3.10)	4 (5.33)		
	B1		1 (0.57)	0 (0.00)	1 (1.03)	0 (0.00)		
	B2		22 (12.50)	14 (12.81)	11 (11.34)	4 (5.53)		
	B3		24 (13.64)	17 (14.91)	14 (14.43)	6 (8.00)		
	C1		2 (1.14)	0 (0.00)	0 (0.00)	1 (1.33)		
	C2		9 (5.11)	7 (6.14)	3 (0.93)	3 (4.00)		
	C3		84 (47.72)	68 (59.65)	58 (59.80)	55 (73.33)		

*n*—number of participants; mm^3^—cube millimeter; ^1^—Kruskal–Wallis Test; ^2^—Chi-Squared Test.

**Table 6:** Comparison of immunological and virological parameters between ART-naive and ART-experienced patients across different study groups.

Parameters		CD4 T-Cell Counts (Cells/mm^3^)	CD8 T-Cell Counts (Cells/mm^3^)	CD4/CD8 Ratio	HIV-1 RNA Viral Load (Copies/mL)
		*p* Value Mann–Whitney U Test			
HIV *n* = 176	Naive	0.818	0.393	0.434	0.327
	Experienced				
HIV/HBV *n* = 114	Naïve	0.625	0.450	0.522	0.628
	Experienced				
HIV/HCV *n* = 97	Naïve	0.055	0.098	0.629	0.539
	Experienced				
HIV/HBV/HCV *n* = 75	Naïve	0.225	0.183	0.563	0.614
	Experienced				

**Table 7:** Pearson correlation between the recent values of CD4 count cell and HIV1-RNA and different variables for patients in HIV mono-infected group.

Variables	CD4 Count (Cells/mm^3^)		HIV1-RNA Load (Copies/mL)	
	Pearson’s r	*p* Value	Pearson’s r	*p* Value
Age	0.156	0.038	0.099	0.189
Age at diagnosis	0.054	0.478	0.052	0.491
No. of years of illness	0.147	0.051	0.063	0.408
No. of years of therapy	0.135	0.073	0.048	0.529
First RNA (copies/mL)	0.023	0.762	−0.023	0.760
CD4 at diagnosis (cells/mm^3^)	0.067	0.373	−0.096	0.204
Nadir CD4	−0.035	0.643	−0.071	0.348
No. of ARV regimens	−0.086	0.256	0.056	0.462

No.—number; mm^3^—cube millimeter.

**Table 8:** Pearson correlation between the recent values of CD4 cells and HIV1-RNA and different variables for patients in HIV/HBV coinfection group.

Variables	CD4 Count (Cells/mm^3^)		HIV1-RNA Load (Copies/mL)	
	Pearson’s r	*p* Value	Pearson’s r	*p* Value
Age	0.063	0.506	−0.057	0.546
Age at diagnosis	0.172	0.067	0.021	0.824
Number of years of illness	−0.192	0.041	−0.109	0.247
Number of years of therapy	−0.197	0.035	−0.044	0.645
First HIV1-RNA load (copies/mL)	0.100	0.289	−0.031	0.743
CD4 at diagnosis (cells/mm^3^)	0.062	0.512	0.136	0.148
Nadir CD4 (cells/mm^3^)	0.044	0.645	0.088	0.352
No. of ARV regimens	−0.128	0.176	−0.083	0.379

**Table 9:** Pearson correlation between the recent values of CD4 count cell and HIV1-RNA load copies/mL and different variables for patients in HIV/HCV coinfection group.

Variables	CD4 Count (Cells/mm^3^)		HIV1-RNA Load (Copies/mL)	
	Pearson’s r	*p* Value	Pearson’s r	*p* Value
Age	0.176	0.087	0.053	0.611
Age at diagnosis	0.074	0.475	0.019	0.853
Number of years of illness	0.115	0.266	0.039	0.708
Number of years of therapy	−0.003	0.979	0.099	0.339
First RNA (copies/mL)	0.008	0.937	0.009	0.929
CD4 at diagnosis (cells/mm^3^)	0.129	0.209	−0.069	0.502
Nadir CD4 (cells/mm^3^)	0.268	0.008	−0.103	0.318
No. of ARV regimens	−0.038	0.714	0.069	0.506

**Table 10:** Pearson correlation between the recent values of CD4 count cell and HIV1-RNA load copies/mL and different variables for patients in HIV/HBV/HCV triple-infection group.

Variables	CD4 Count (Cells/mm^3^)		HIV1-RNA Load (Copies/mL)	
	Pearson’s r	*p* Value	Pearson’s r	*p* Value
Age	0.020	0.864	−0.053	0.653
Age at diagnosis	0.063	0.592	−0.130	0.268
Number of years of illness	−0.083	0.480	0.153	0.191
Number of years of therapy	−0.217	0.041	0.046	0.694
First HIV1-RNA load (copies/mL)	−0.044	0.707	−0.072	0.538
CD4 at diagnosis (cells/mm^3^)	−0.007	0.949	0.229	0.049
Nadir CD4 (cells/mm^3^)	0.091	0.436	0.084	0.472
No. of ARV regimens	−0.112	0.340	−0.007	0.953

**Table 11:** Descriptive statistics of hepatic function.

Parameters	HIV *n* = 176	HIV/HBV *n* = 114	HIV/HCV *n* = 97	HIV/HBV/HCV *n* = 75	Statistics	*p* Value
ALT (IU/L)	53.158 ± 48.904	25.256 ± 8.873	27.823 ± 21.787	17.840 ± 14.321	198.488 ^1^	<0.001 ^1^
AST (IU/L)	35.938 ± 26.507	28.693 ± 9.372	34.427 ± 27.151	36.587 ± 48.460	5.081 ^1^	0.166 ^1^
AST/ALP	0.745 ± 0.198	1.141 ± 0.150	1.280 ± 0.214	2.009 ± 0.589	344.494 ^1^	<0.001 ^1^
GGT (IU/L)	20.616 ± 10.796	34.851 ± 11.334	46.396 ± 14.599	167.947 ± 294.338	344.211 ^1^	
Amylase (IU/L)	48.723 ± 11.250	70.667 ± 7.499	77.021 ± 11.283	125.813 ± 44.156	343.869 ^1^	
Lipase (IU/L)	65.689 ± 66.712	125.447 ± 57.348	190.521 ± 86.039	642.987 ± 209.566	336.872 ^1^	
Direct bilirubin (mg/dL)	0.171 ± 0.062	0.254 ± 0.061	0.305 ± 0.065	0.563 ± 0.372	303.898 ^1^	
Indirect bilirubin (mg/dL)	0.210 ± 0.095	0.374 ± 0.083	0.449 ± 0.099	1.145 ± 0.968	332.998 ^1^	
Total bilirubin (mg/dL)	0.418 ± 0.112	0.612 ± 0.078	0.683 ± 0.128	1.504 ± 1.096	336.035 ^1^	
ALP (IU/L)	56.045 ± 11.983	76.123 ± 8.226	83.938 ± 13.099	133.360 ± 50.955	344.105 ^1^	

*n*—number of participants; AST—aspartate transaminase; ALT—alanine aminotransferase; GGT—gamma-glutamyl transferase; ALP—alkaline phosphatase; IU—international units; ^1^—Kruskal–Wallis Test.

**Table 12:** Dunn’s Post Hoc Comparisons between studies.

Parameter	HIV vs. HIV/HBV/HCV	HIV vs. HIV/HCV	HIV vs. HIV/HBV	HIV/HBV/HCV vs. HIV/HBV	HIV/HBV/HCV vs. HIV/HCV	HIV/HCV vs. HIV/HBV
			*p* Value			
ALT	<0.001	<0.001	<0.001	<0.001	<0.001	0.335
AST	0.089	0.125	0.070	0.909	0.794	0.866
AST/ALT	<0.001	<0.001	<0.001	<0.001	<0.001	0.008
GGT						0.009
Amylase						0.010
Lipase						0.109
Direct bilirubin						<0.001
Indirect bilirubin						0.002
Total bilirubin						0.011
ALP						0.009

AST—aspartate transaminase; ALT—alanine aminotransferase; GGT—gamma-glutamyl transferase; ALP—alkaline phosphatase.

**Table 13:** Statistical results of the odds ratio analysis.

Parameters	HIV/HBV/HCV vs. HIV	HIV/HBV vs. HIV	HIV/HCV vs. HIV
ALT	OR = 8.2, *p* < 0.001 OR∈[2.86; 23.61]	OR = 52.3, *p* < 0.001 OR∈[7.12; 384.09]	OR = 4.47, *p* < 0.001 OR∈[2.1; 9.5]
AST	OR = 2.29, *p* = 0.012 OR∈[1.18; 4.43]	OR = 1.97, *p* = 0.014 OR∈[1.14; 3.41]	OR = 1.66, *p* = 0.048 OR∈[1.09; 2.93]
AST/ALT ratio	OR > 50, *p* < 0.001 OR∈[20.06; 662.2]	OR > 50, *p* < 0.001 OR∈[45.95; 702.47]	OR > 50, *p* < 0.001 OR∈[39.49; 789.5]
GGT	OR > 50, *p* < 0.001 OR∈[23.13; 662.26]	OR = 11.74, *p* < 0.001 OR∈[4.39; 31.39]	OR > 50, *p* < 0.001 OR∈[35.96; 265.3]
Amylase	OR > 50, *p* < 0.001 OR∈[30.1; 835.8]	OR > 50, *p* < 0.001	OR > 50, *p* < 0.001
Lipase	OR > 50, *p* < 0.001 OR∈[14.38; 386.82]	OR > 50, *p* < 0.001 OR∈[21.87; 584.59]	R > 50, *p* < 0.001 OR∈[18.4; 493.31]
Direct bilirubin	OR = 12.07, *p* = 0.029 OR∈[1.573; 254.58]	OR > 50, *p* < 0.001	OR > 50, *p* < 0.001
Indirect bilirubin	OR > 50, *p* < 0.001 OR∈[8.9; 250.41]	OR > 50, *p* < 0.001	OR > 50, *p* < 0.001
Total bilirubin	OR > 50, *p* < 0.001 OR∈[13.5; 375.99]	OR > 50, *p* < 0.001	OR > 50, *p* < 0.001
ALP	OR > 50, *p* < 0.001 OR∈[6.28; 179.46]	OR > 50, *p* < 0.001	OR > 50, *p* < 0.001

AST—aspartate transaminase; ALT—alanine aminotransferase; GGT—gamma-glutamyl transferase; ALP—alkaline phosphatase.

## Data Availability

Data are available from the first author.

## Abbreviations

The following abbreviations are used in this manuscript:

ABC	Abacavir
ALP	Alkaline phosphatase
ALT	Alanine aminotransferase
ARV	Antiretroviral
AST	Aspartate transaminase
B	Bictegravir
COBI	Cobicistat
DILI	Drug-induced liver damage
DRV	Darunavir
DTG	Dolutegravir
EFZ	Efavirenz
EI	Entry inhibitor
ETV	Etravirine
FTC	Emtricitabine
FDA	U.S. Food and Drug Administration
GGT	Gamma-glutamyl transferase
HAART	Highly active antiretroviral therapy
HBsAg	Hepatitis B surface antigen
HBV	Hepatitis B virus
HCV	Hepatitis c virus
HIV	Human immunodeficiency virus
II	Integrase inhibitor
INSTI	Integrase strand transfer inhibitors
IU	International units
3TC	Lamivudine
LFT	Liver function test
MSM	Men who have sex with men
NAFLD	Non-alcoholic fatty liver disease
NIID MB	National Institute of Infectious Diseases “Prof. Dr. Matei Balș”
NNRTI	Non-nucleoside reverse transcriptase inhibitor
NRTI	Nucleoside reverse transcriptase inhibitor
PCR	Real-time polymerase chain reaction
PI	Protease inhibitor
PLWH	People living with HIV
PWIDs	People who inject drugs
RAL	Raltegravir
RTV	Ritonavir
RNA	Ribonucleic acid
TAF	Tenofovir alafenamide
TDF	Tenofovir disoproxil fumarate
ZDV	Zidovudine
